# Nutrition and depression at the forefront of progress

**Published:** 2012-12-25

**Authors:** TA Popa, M Ladea

**Affiliations:** * 3rd Psychiatric Department, “Alexandru Obregia” Clinical Hospital of Psychiatry, Bucharest, Romania; ** “Carol Davila” University of Medicine and Pharmacy, Bucharest, Romania; 3rd Psychiatric Department, “Alexandru Obregia” Clinical Hospital of Psychiatry, Bucharest, Romania

**Keywords:** nutritional factors, diet, depression, neurotransmitters

## Abstract

Depression is a debilitating disorder estimated to become the second cause of morbidity worldwide by the year 2020. The limited efficacy of antidepressant therapy, as well as the major negative consequences of this disorder, has stimulated additional research in order to determine possible adjunctive treatments. There is mounting evidence linking dietary patterns to major depression development.

This article presents some of the most significant findings concerning the role of nutrition in major depressive disorder. Although more focused and clear results are needed, the correlation between nutrition and mental health is gaining attention. Now, there is evidence supporting the importance of nutrition in maintaining good mental health. We emphasize multiple findings that support adherence to healthy dietary patterns, taking into account that the production of neurotransmitters need, among others, right amounts of nutrients, a lot of which can only be supplied through diet. Not only certain nutrients are needed for proper brain functioning, but also others can be harmful, promoting depression. The Mediterranean diet has been linked to a low prevalence of depression while fast-food consumption has been found to increase the risk of developing and aggravating this disorder, hence the need for nutritional interventions.

From the perspective of discovering modifiable risk factors, the role of nutrition in psychiatry could be more important than it was initially considered.

Major depressive disorder (MDD) is one of the main causes of disability in developed countries as well as in low and medium income countries. Depression is considered to increase 23-fold the disability risk in comparison to the general population. 

The World Health Organization (WHO) estimates that by the year 2020 depression will be the second cause of morbidity worldwide. Around 150 million people suffer from depression worldwide and this number has increased in recent years [**1**]. This means that it is one of the main global causes of disability-adjusted life year. The prevalence of this disorder is high: one out of 7 individuals will suffer a depressive episode during his or her lifetime. 

To our knowledge, the most recent study [**2**] concerning the prevalence of depression gathered data reported by 18 countries. The results found that the highest prevalence of MDD was recorded in France (21%) and the United States of America (USA, 19%). It is worth mentioning that, overall, the highest prevalence was reported by high-income countries (15%), compared to the one found in low-income areas (11%).

 Numerous researchers attempted to explain the high or low rates of depression in different countries and the possible etiological factors. Although many obstacles were identified when interpreting the results, such as cultural, social, and economic factors, as well as different evaluation tools and diagnostic criteria used, most studies show that sex, age, and marital status can be associated with the risk of developing MDD. Women seem to be twice as likely to become depressed [**3**], and separated or divorced individuals may have greater susceptibility [**4**]. Also the level of stress, recent life events, alcohol and drug abuse, lack of social support, financial and professional status, as well as family history were linked to MDD development [**6-8**].

 Often under-diagnosed, depression can have major consequences, such as personal, professional and social dysfunction, premature mortality, and most important - suicide. All these outcomes are responsible for the important burden and the increased costs generated by MDD. In the USA alone, the annual costs of depression raise up to 44 billion dollars [**9**].

 Research shows that only one fourth of those suffering from MDD have access to specific treatment options, such as antidepressant medication and psychotherapy. The risk of relapse is high and most often the course of MDD is recurrent. 

Unfortunately, only around 60% of those receiving adequate treatment benefit from clinical improvement. This relatively low percentage raises even more problems since the individuals who do not benefit from antidepressant medication have very few options left (i.e. psychological interventions, social support).

 The action mechanism of antidepressant medication is not yet fully understood. What we know so far is that it can adjust the level of neurotransmitters involved in MDD development. Among the most important effects of this treatment is the serotonin and also noradrenalin reuptake inhibitory action. Serotonin is known to regulate mood, emotion, sleep, and appetite thus controlling many psychic and behavioural functions. Serotonin reuptake inhibitors (SSRIs) are recommended as the first-line of treatment in MDD.

 Another effective antidepressant medication class is represented by monoamine oxidase inhibitors (MAOIs), but they are less prescribed in clinical practice due to their strict dietary precautions (i.e. thiamine-induced hypertension) and their many drug interactions. MAOIs prevent the degradation of monoamine neurotransmitters, such as serotonin, melatonin, epinephrine, norepinephrine, dopamine and others.

 Given the important personal, social, professional, and economic impact of MDD sufferers, as well as the low treatment response, the latency of antidepressant action, treatment adherence issues, and potential side effects of antidepressant medication, there is a great need for adjunctive treatment.

Recent research has focused on the role of nutrition in the management of depression. We know that the production of neurotransmitters needs adequate amounts of nutrients. Among these nutrients, we mention amino acids (tryptophan, tyrosine, and glutamine), minerals (zinc, copper, iron, magnesium), and B vitamins (B6, B12, folic acid). These are found in whole grains (zinc, copper, magnesium), eggs, cheese, yogurt (tyrosine, glutamine, zinc, magnesium), beans, vegetables, especially green leaf ones, broccoli, cabbage, spinach, corn, fish, poultry, etc.

 Most of the food mentioned above is part of the Mediterranean diet, known for the nutritional balance it provides. Little is known for now about the role that diet plays in the development of depressive disorders. Previous studies suggest that certain nutrients may be important in preventing the development of MDD. These include group B vitamins, omega-3 fatty acids, and olive oil. Furthermore, a healthy diet as is the Mediterranean type (rich in fruits, vegetables, fish and cereals, but low in meat and dairy products) has been associated with a lower risk of developing depression [**10**].

 On the other hand, a diet that is high in refined carbohydrates and sugars is a common factor in depressive illness. Alcohol can also have a severe depressant effect.

In the last decades, deficiencies in neurotransmitters such as serotonin, dopamine, noradrenalin, and γ-aminobutyric acid (GABA) were associated with MDD [11-15].

 Several studies show that amino acids such as tryptophan, tyrosine, and phenylalanine could be helpful in treating depression [**16-19**]. Considering this evidence, it can be implied that restoring serotonin levels may decrease the symptoms of depression precipitated by serotonin deficiencies. The medication aside, this goal can be achieved through adherence to a diet that is high in tryptophan. In addition, the amino acid tyrosine and sometimes its precursor phenylalanine are converted into dopamine and norepinephrine.

In a 2009 study [**20**], the final phase included 3,486 adult men and women between 33 and 55 years of age who were categorized as having either a “whole food” (rich in fish, fruits and vegetables) or “processed food” (rich in processed meat, chocolates, sweet desserts, pies, condiments, fried food, refined cereals and high fat dairy products) dietary pattern. After adjusting for gender, age, energy intake, marital status, medication, other medical conditions etc., participants with the highest intake of ‘whole food’ were the least likely to be depressed compared to those with the lowest adherence to this dietary pattern, based on the Center for Epidemiologic Studies- Depression Scale (CES-D scale). Therefore, a diet rich in processed food may increase the risk of depression. There is also the possibility that those already at greater risk of depression may tend to consume more processed food. Although the authors concluded that the lower the quality of the diet, the higher the risk of depression, cause and effect cannot be yet established. The role of each individual food item in the risk of depression would need to be assessed for a causality relationship to be demonstrated.

In addition, the link between fast food and depression has recently been confirmed. The large European study followed and analyzed the diet and lifestyle of over 12,000 volunteers free of depression over a period of six years [**21**]. Participants with an elevated consumption of trans-fats (present in pastries and fast food) had up to a 48 percent increase in the risk of depression when compared to participants who did not consume these fats. Later on, after assessing the role of polyunsaturated fats (composed of larger amounts of fish and vegetable oils) and olive oil in MDD development, these products were found to be "associated with a lower risk of suffering of depression." The authors believe that the global rise in MDD sufferers in recent years could be attributable "to radical changes in the sources of fats consumed in Western diets, where we have substituted certain types of beneficial fats – polyunsaturated and monounsaturated in nuts, vegetable oils and fish – for the saturated and trans-fats found in meats, butter and other products such as mass-produced pastries and fast food."

 In consistency with the findings mentioned above, the so-called “Western” dietary pattern (rich in saturated fatty acids and trans-fatty acids and common in Northern Europe and USA) has been implicated as a relevant risk factor for developing depression [**20,22**].

 Aside from the dietary patterns taken as a whole, the role of different nutrients in the risk of developing depression has been studied intensively in recent years.

 The brain is one of the organs with the highest level of lipids. Brain lipids, composed of fatty acids, are structural constituents of membranes. It has been estimated that gray matter contains 50% polyunsaturated fatty acids out of which about 33% belong to the omega-3 family. Being essential fatty acids, meaning that they cannot be synthesized in the body, they must be supplied through diet.

 In one of the first coherent experimental demonstrations of the effect of dietary substances on the structure and function of the brain researchers studied omega-3 fatty acids. Experiments were first carried out on x-vivo cultured brain, then on in vivo brain cells, and finally on physicochemical, biochemical, physiological, neurosensory, and behavioural parameters. Although conducted only on fatty acids present in formula milks for infants, the results indicated that the nature of polyunsaturated fatty acids (in particular omega–3) could determine the visual, cerebral, and intellectual abilities [**23**]. 

 The two omega-3 fatty acids, eicosapentaenoic acid (EPA) and docosahexaenoic acid (DHA), found in fish oil, have been found to have antidepressant effects. DHA may be of more interest, since the body ultimately converts EPA into DHA, but there are limited clinical studies on pure DHA. Another EPA bioconversion pathway also involves the production of prostaglandins, thromboxanes and leukotrienes. Many of the proposed mechanisms of this conversion involve neurotransmitters. For instance, antidepressant effects may be due to bioconversion of EPA to leukotrienes, prostaglandins, and other chemicals required by the brain and implicated in the body’s immune responses. Whichever may be the case, evidence gathered throughout the years has shown that omega-3 fatty acids can help treat depression. 

 A comprehensive review [**24**] discusses epidemiological and treatment studies supporting the antidepressant role of omega-3 supplementation. Deficits in omega-3 fatty acids have been identified as a contributing factor to mood disorders and offer a potential rational treatment approach. Case-control studies have shown that patients suffering with depression have significantly lower levels of omega-3 [**25**] and clinical trials have indicated the effectiveness of omega-3 as adjunctive treatment for major depression [**20,22**].

 Accumulating evidence from demographic studies also indicates a link between high fish consumption and low incidence of mental disorders; this lower incidence rate being the direct result of omega-3 fatty acid intake [**28**]. Experimental studies have revealed that diets low in omega-3 polyunsaturated fatty acids (PUFAs) lead to considerable disturbance in neural function [**29**].

There is biologically plausible evidence to suggest that omega-3 PUFAs might play a role as adjunctive therapy for depression, though much research is required to determine the most effective omega-3 PUFA (EPA, DHA or a mixture of both) and the most effective dose.

Folate and vitamin B12 deficiencies have also been linked to depression [**50**]. The author suggests that patients treated with 0.8 mg of folic acid per day or 0.4 mg of vitamin B12 per day will exhibit decreased depression symptoms. Folate's vital role in brain metabolic processes has been recognized by many researchers who have noted that depressive symptoms are the most common neuropsychiatric manifestation of folate deficiency [**30**].

 It has been observed that patients with depression have blood folate levels approximately 25% lower than healthy controls. One study compared the effects of placebo or folate intake in addition to fluoxetine (an SSRI) treatment. Overall, there was a significantly greater improvement in the fluoxetine plus folic acid group [**31**].

 Low levels of folate have also been identified as an important predisposing factor of poor outcome in patients following antidepressant therapy. Another controlled study has shown that 500 mcg of folic acid enhanced the effectiveness of antidepressant medication [**32**].

 Furthermore, according to a study [**33**], supplementation of nine vitamins, 10 times in excess of usual recommended dietary allowance (RDA) for one year may improve mood. It is worth mentioning that these changes in mood after a year occurred even though the blood status of nine vitamins reached a plateau after 3 months.

 Many neurotransmitters also depend on the availability of amino acids. Eight out of 20 amino acids have to be supplied through diet, for they cannot be synthesized in the human body. These are the essential amino acids. A high quality protein diet contains all essential amino acids. Food rich in high quality protein include meat, milk and other dairy products, and eggs. Plant proteins such as beans, peas, and grains may be low in one or two essential amino acids. Protein intake and in turn the individual amino acids can affect the brain functioning and mental health. 

 A Canadian study showed that l-tyrosine depletion in healthy women could determine a decrease in catecholamine-dependent neurotransmission, thus increasing the risk of mood and anxiety disorders. Due to its role in neuronal stimulation, tyrosine can directly affect mood and cognitive functions [**34**]. Low blood levels of tyrosine were found in some MDD patients [**35**].

 Mineral deficiencies have also been linked to MDD development, although much of this relationship awaits more extended research. Selenium and zinc are two of the minerals that aroused scientific interest.

 A review identified five studies which indicate that low selenium intake is associated with lowered mood status [**36**].

 Other studies have shown that zinc levels are lower in individuals with clinical significant depression [**37**]. Furthermore, research shows that the intake of oral zinc can enhance the effectiveness of antidepressant therapy [**38**].

 Zinc also protects the brain cells against the potential damage caused by free radicals. 

 Also, antioxidants (such as vitamin C and E) can fight against the damage free radicals induce.

 It is established that many nutritional deficiencies have been associated with an increased risk of depression. At the same time, an excess of nutrients, particularly refined sugar, has also been linked to MDD.

 A large study conducted in 6 countries established a highly significant correlation between sugar consumption and the prevalence of depression [**39**]. This relationship is yet unclear but a high intake of sugar may interfere with the production of neurotransmitters.

 Current research in psychoneuroimmunology is concentrating on the possibility of interconnected pathways that can constitute the basis for a clearer understanding of the link between nutritional intake, central nervous system, and immune function thereby influencing an individual's mental health status. Several recent studies have implicated inflammation in the physiopathology of depression [**40,41**].

 Diets high in refined starches, sugar, and saturated and trans-fatty acids, poor in natural antioxidants and fiber from fruits, vegetables, and whole grains, and poor in omega-3 fatty acids may cause an activation of the inflammatory pathways [**42**]. 

 This could mean that diets that promote inflammation could fuel depressive symptoms. Thus, diet influences inflammation, and dietary-related inflammation may in turn increase depression and depression can in turn advance inflammation.

In contrast, the omega-3 fatty acids, found in fish, fish oil, walnuts, wheat germ, and some dietary supplements such as flax seed products can reduce inflammation [**43,44**].

 On the other side, many studies suggest that depression and stressful events can determine less healthy food choices, although there may be a greater risk related to being female, overweight, and scoring high on dietary restraint [**45,46**]. For instance, one large study found that stress and depression were associated with less fresh fruit consumption as well as greater snack food intake [**47**]. Female students in Germany, Poland, and Bulgaria who reported more perceived stress ate more sweets and fast food, and fewer fruits and vegetables than those who were less stressed [**48**]. Another study found that men decreased their vegetable intake following divorce or bereavement, and increased consumption after remarriage [**49**]. Therefore, stress and depression promote less healthy food choices that can activate the inflammatory pathways. Stress complicates the consequences even more by possibly promoting adverse and maladaptive metabolic responses to unhealthy diets.

The findings mentioned in this article may shed more light on the therapeutic value of dietary intervention among medical personnel addressing depression and other psychological disorders.

As explained so far, depression is a major public health issue that needs adjunctive therapy options. It is undeniably linked to nutrition, as suggested by the mounting evidence by research in neuropsychiatry. 

An interesting observation is that the nutrition of individuals suffering from MDD is far from adequate. Multiple factors ranging from poor appetite to unhealthy food choices could be responsible for an inappropriate diet.

 Among all the potential risk factors and triggers linked to MDD, nutrition is possibly the most basic factor and may be the easiest to modify. Nutrition certainly deserves more attention and assessment, particularly among individuals suffering from mental disorders.

 An adequate intake of good calories, healthy proteins, omega-3 fatty acids and all essential minerals is of utmost importance in maintaining good mental health.

 However, a correct assessment of the impact of nutrition on the development and course of depression is difficult to attain. The role of each nutrient needs to be studied separately in order to obtain results that are more accurate. Further research also needs to determine the best recommended doses of nutritional supplements that could bring the greatest benefit for mentally ill patients. It is worth mentioning that no matter the doses of nutritional supplements, their use can only be considered as adjunctive treatment and cannot substitute antidepressant medication.

Until more research will be available, health care practitioners could work towards improving patients’ nutritional status and behaviour. Dietary supplements can also be recommended by physicians, based on previous and current efficacious trials and the doses adjusted depending on the obtained results.
